# 5-Aminoimidazole-4-carboxamide ribonucleoside-induced autophagy flux during differentiation of monocytic leukemia cells

**DOI:** 10.1038/cddiscovery.2017.66

**Published:** 2017-10-02

**Authors:** Vilma Dembitz, Hrvoje Lalic, Dora Visnjic

**Affiliations:** 1 Department of Physiology and Croatian Institute for Brain Research, School of Medicine, University of Zagreb, Salata 12, Zagreb 10 000, Croatia

## Abstract

Pharmacological modulators of AMP-dependent kinase (AMPK) have been suggested in treatment of cancer. The biguanide metformin and 5-aminoimidazole-4-carboxamide ribonucleoside (AICAR) have been reported to inhibit proliferation of solid tumors and hematological malignancies, but their role in differentiation is less explored. Our previous study demonstrated that AICAR alone induced AMPK-independent expression of differentiation markers in monocytic U937 leukemia cells, and no such effects were observed in response to metformin. The aim of this study was to determine the mechanism of AICAR-mediated effects and to test for the possible role of autophagy in differentiation of leukemia cells. The results showed that AICAR-mediated effects on the expression of differentiation markers were not mimicked by A769662, a more specific direct AMPK activator. Long-term incubation of U937 cells with AICAR and other differentiation agents, all-*trans*-retinoic acid (ATRA) and phorbol 12-myristate 13-acetate, increased the expression of the autophagy marker LC3B-II, and these effects were not observed in response to metformin. Western blot and immunofluorescence analyses of U937 cells treated with bafilomycin A1 or transfected with mRFP-GFP-LC3 proved that the increase in the expression of LC3B-II was due to an increase in autophagy flux, and not to a decrease in lysosomal degradation. 3-Methyladenine inhibited the expression of differentiation markers in response to all inducers, but had stimulatory effects on autophagy flux at dose that effectively inhibited the production of phosphatidylinositol 3-phosphate. The small inhibitory RNA-mediated down-modulation of Beclin 1 and hVPS34 had no effects on AICAR and ATRA-mediated increase in the expression of differentiation markers. These results show that AICAR and other differentiation agents induce autophagy flux in U937 cells and that the effects of AICAR and ATRA on the expression of differentiation markers do not depend on the normal levels of key proteins of the classical or canonical autophagy pathway.

## Introduction

Several studies have suggested that drugs that modulate activity of AMP-activated kinase (AMPK) have potential for the treatment and prevention of cancer. The biguanide metformin, a widely used drug for the treatment of type 2 diabetes, and 5-aminoimidazole-4-carboxamide ribonucleoside (AICAR, acadesine) have been reported to exert antiproliferative effects in various solid tumors and hematological malignancies, but their role in differentiation has been less explored.^1,2^ Although both drugs activate AMPK, an evolutionary conserved serine/threonine kinase that is activated whenever the energy level in the cell is low and the ratio of AMP to ATP increased, an increasing number of studies demonstrate that majority of beneficial effects of metformin and AICAR are actually AMPK-independent.^3,4^ Our recent study demonstrated that AICAR alone induced the expression of cell surface markers associated with mature monocytes and macrophages in monocytic U937 cells.^5^ However, no significant increase in the expression of differentiation markers was observed in U937 cells treated with metformin alone, although the effects on proliferation and survival were similar to the ones observed in the presence of AICAR.

It is still unknown which are the mechanisms responsible for AICAR-mediated effects in acute myeloid leukemia (AML) cell lines. Within the cell, AICA-ribonucleoside could be phosphorylated by adenosine kinase into AICA-ribonucleotide or ZMP, which is an analog of 5′-AMP; ZMP then binds to *γ* regulatory subunit and activates AMPK in a similar manner to AMP.^6^ Although we detected time- and dose-dependent increase in the level of Thr-phosphorylated AMPK, a significant decrease in AMPK expression that was achieved by using commercially available siRNA sequences in U937 cells had no significant effects on the AICAR-mediated effects on the number of viable cells or the expression of differentiation markers.^5^ Therefore, present studies are aimed to determine the mechanism responsible for beneficial effects of AICAR in AML cells and to further elucidate signaling mechanisms responsible for differentiation of U937 cells in response to other inducers.

In chronic myelogenous leukemia (CML) cell lines, AMPK-independent cell death induced by AICAR involved autophagy, the major intracellular pathway for the selective degradation of cytoplasmic organelles and long-lived proteins.^7^ Although initially described as a protective mechanism that allowed cells to survive in the absence of nutrients, the autophagy has been recently implicated in several other conditions, including cell death, neurodegeneration, immunity, cancer and differentiation.^8,9^ Autophagy is mediated by autophagy-related (*ATG*) genes that are evolutionary conserved from yeasts to mammals; a key initiation signal is provided by a complex consisting of Beclin 1 (or Atg6), class III phosphatidylinositol 3-kinase (PI3KC3 or Vps34), Vps15 and Atg14. In unstressed cells, autophagy is inhibited by mammalian target of rapamycin complex 1 (mTORC1). Therefore, inhibition of mTOR by either rapamycin or AMPK activation promotes autophagy.^8^ Recent studies pointed to the role of autophagy in differentiation of some leukemia cell lines, including all-*trans*-retinoic acid (ATRA)-induced differentiation of acute promyelocytic leukemia NB4 cells,^10–12^ vitamin D_3_-mediated differentiation of myeloblastic HL-60 cells^13^ and megakaryocytic differentiation of CML K562 cells in response to phorbol 12-myristate 13-acetate (PMA) or lapatinib.^14,15^

In the present study, we tested for the possible role of autophagy in AICAR-mediated differentiation of monocytic U937 leukemia cells. The results of the study showed that long-term incubation of U937 with AICAR and other inducers of differentiation induced autophagy flux that was not observed in cells treated with metformin. However, AICAR-mediated increase in the expression of differentiation markers did not depend on the presence of key proteins of canonical autophagy pathway.

## Results

**Figure 1:**
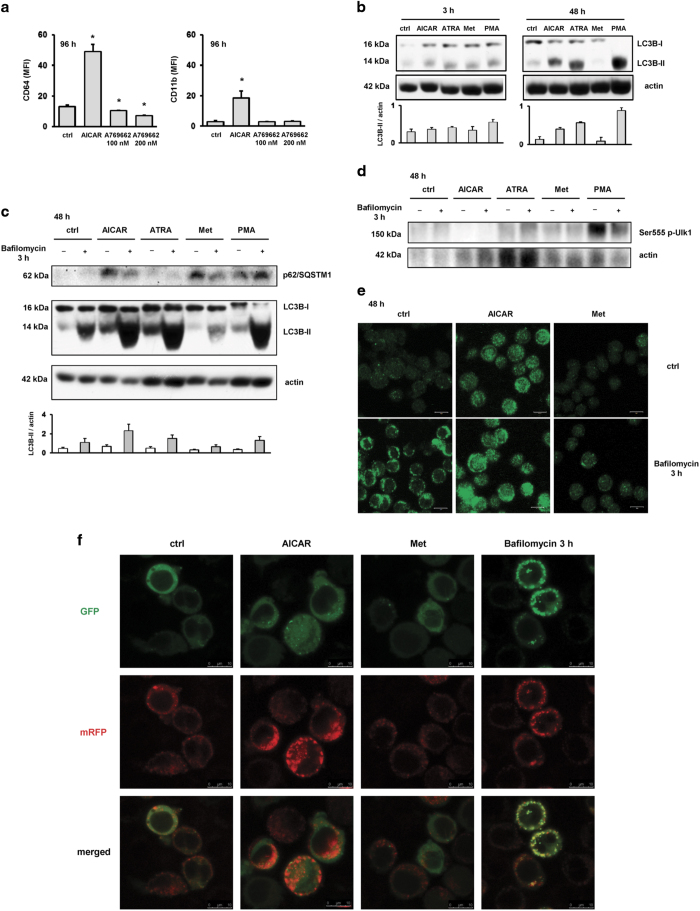
AICAR and other differentiative agents induce autophagy in U937 cells. (**a**) U937 cells were treated with AICAR (0.5 mM) and direct AMPK agonist A769662 (100 or 200 nM) for 96 h and the expression of differentiation markers was analyzed by flow cytometry. Results are mean±S.E.M. of three independent experiments. *P<0.05. (**b**) AICAR and other differentiation inducers increase the expression of LC3B-II, an autophagy marker. Total cell lysates were isolated 3 or 48 h after addition of AICAR (0.5 mM), ATRA (1 *μ*M), metformin (15 mM) or PMA (50 nM) and analyzed by western blot. Representative immunoblot from three independent experiments is shown. Blots were scored by densitometry and graphs represent mean±S.E.M. of LC3B-II/actin ratios from three independent experiments. (**c**) Cells were treated for 48 h as indicated above and bafilomycin (50 nM) was added during the last 3 h of incubation for the assessment of autophagic flux. Representative immunoblot from three independent experiments is shown. Graph represents mean±S.E.M. of LC3B-II/actin ratios from three independent experiments. (**d**) AICAR does not increase phosphorylation of Ulk1 on Ser555. Cells were treated as indicated above and representative immunoblot from three independent experiments is shown. (**e**) Immunolabeling of U937 cells treated with AICAR (0.5 mM) and metformin (15 mM) demonstrates that AICAR induces formation of LC3B puncta. Bafilomycin (50 nM) was added as described above and used for the assessment of autophagic flux. Representative images of confocal microscropy from three independent experiments are shown. Images are ×63 with ×2 times digital zoom, scale bar=10 *μ*m. (**f**) Analysis of autophagic flux in AICAR-treated U937 cells stably transfected with mRFP-GFP-LC3B. Cells were incubated for 48 h with AICAR (0.5 mM) or metformin (15 mM). Bafilomycin (50 nM) was added during the last 3 h of incubation. Representative images of confocal microscopy from two independent experiments performed in duplicates are shown. Images are ×63 with ×4 times digital zoom, scale bar=10 *μ*m.

To corroborate the finding that AICAR-mediated effects are AMPK-independent, we first tested the effects of A769662, a more specific direct AMPK activator, on the proliferation and differentiation of monocytic U937 cells. Although the incubation with A769662 for 96 h reduced the number of viable U937 cells comparable to the effect of AICAR (data not shown), there were no effects on the level of either CD64 or CD11b (Figure 1a). Therefore, we concluded that AICAR-mediated effects in leukemia cell lines cannot be mimicked by a more specific AMPK activator.

To test for the possible role of AICAR as an autophagy inducer, we measured the level of LC3B, which is widely used as a reporter of autophagosome formation.^16^ During autophagy, LC3B is specifically cleaved to form LC3-I, and cytosolic LC3-I is lipidated to the autophagosome membrane-bound LC3-II form that migrates faster in SDS-PAGE. For western blot analyses, U937 cells were first incubated in the presence of AICAR, other differentiation inducers (ATRA, PMA) and metformin. Total cell lysates were isolated 3 and 48 h after the addition of agents and analyzed for the expression of LC3B. As shown in Figure 1b, all agents slightly increased the level of LC3B-II after 3 h. However, after 48 h, the ratio of LC3B-II to actin was significantly increased in cells treated with AICAR and other differentiation agents, while there was no increase in cells treated with metformin.

The increase in LC3B-II levels could be related to either enhanced formation of LC3B-II, due to an increase in autophagic activity, or reduced turnover, due to an impairment of the degradation. To distinguish between the two, the cells can be treated by bafilomycin A1 that inhibits H^+^-ATPase and blocks lysosomal degradation so that any further accumulation of LC3B-II-positive autophagosomes would be the evidence of autophagic flux.^16^ As shown in Figure 1c, bafilomycin A1 alone increased the level of LC3B-II and had additive effects on the levels of LC3B-II in cells treated with AICAR and other differentiative agents, which indicated an increase in autophagic flux. Again, metformin had no such effects; the LC3B-II/actin ratio in cells treated with combination of bafilomycin A1 and metformin was higher than the ratio in cells treated with metformin, but lower than the ratio in cells treated with bafilomycin A1 alone. As shown in Figure 1c, the level of p62/SQSTM1, an LC3-interacting protein degraded in the autolysosomes, was increased in response to AICAR in accordance with previously published results that demonstrated p62/SQSTM1 upregulation during differentiation of AML cells.^17^ In addition, to check for targets downstream of AMPK, phosphorylation of Ulk1 on Ser555 as a trigger for mTOR-independent autophagy was examined in cells treated with differentiation agents. As shown in Figure 1d, in cells treated with AICAR for 48 h the level of Ulk1 phosphorylated on Ser555 was decreased, further suggesting that AICAR-mediated effects on U937 cells are AMPK-independent.

Additionally, the endogenous LC3B protein was detected by immunofluorescence using the same anti-LC3B antibody (Figure 1e). The analysis by confocal microscopy revealed that immunofluorescent staining changed from diffuse to more punctate in cells treated with AICAR for 48 h, and these changes were more pronounced in cells that were treated in the presence of bafilomycin A1 for last 3 h. Again, no similar effects were observed in cells treated with metformin.

To further verify the increase in autophagic flux, U937 cells were transfected with plasmid containing *LC3B* gene fused with genes encoding mRFP and GFP.^18^ The principle of the method is based on different sensitivities of GFP and mRFP proteins to changes in pH; in acidic environment, green fluorescence of GFP is quenched, while the red fluorescence of mRFP is maintained. As shown in Figure 1f, control U937 cells transfected with mRFP-GFP-LC3B showed some basal level of autophagy. The addition of AICAR increased the number of red dots and these effects were lacking in cells treated with metformin. As expected, bafilomycin A1 increased the level of both green and red fluorescence.

**Figure 2:**
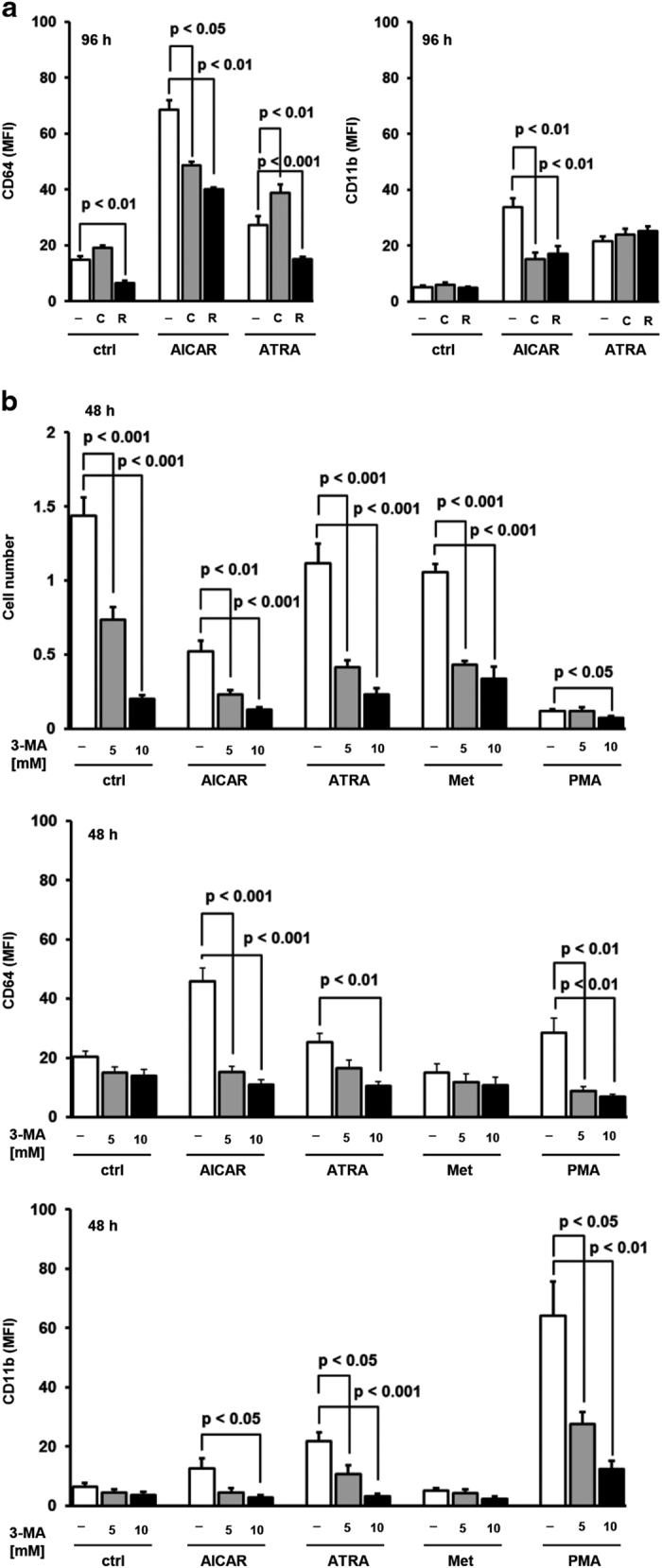
The effect of pharmacological modulators of autophagy on the expression of differentiation markers. (**a**) U937 cells were incubated with AICAR (0.5 mM) and ATRA (1 *μ*M) for 96 h. Chloroquine (25 *μ*M) and rapamycin (20 nM) were added 15 min before addition of differentiation agents. (**b**) U937 cells were incubated with AICAR (0.5 mM), ATRA (1 *μ*M), metformin (15 mM) and PMA (50 nM) for 48 h. 3-MA (5 or 10 mM) was added 15 min before addition of agents tested. The number of viable cells was determined by trypan blue exclusion and the expression of differentiation markers was analyzed by flow cytometry. Results are mean±S.E.M. of at least three independent experiments.

To further test for the role of autophagic flux in differentiative effects of agents, the effects of pharmacological modulators of autophagy were tested in U937 cells. Chloroquine, an inhibitor of lysosomal degradation, has been previously used in concentration ranging from 2.5 to 50 *μ*M to block late autophagy pathway in K562, NB4, primary AML cells and monocytes.^19–22^ In this study, chloroquine was used in concentration of 25 *μ*M in which it exerted only mild cytotoxic effects and no stimulatory effects on the expression of differentiation markers. As shown in Figure 2a, chloroquine inhibited an increase in the expression of CD11b and CD64 in response to AICAR, but had no effects on ATRA-stimulated increase in the expression of CD11b and further increased the expression of CD64.

Although pharmacological inhibition of mTORC1 by rapamycin promoted autophagy in the model of yeasts grown in the presence of nutrients as well as in various tumor cell lines,^23^ different effects of mTOR inhibitors on the expression of differentiation markers have been reported in leukemia cell lines.^24–27^ As shown in Figure 2a, the presence of 20 nM rapamycin prevented an increase in the expression of CD64 and CD11b induced by AICAR. However, rapamycin decreased the level of CD64 but had no significant effects on the level of CD11b in ATRA-treated cells, as previously described.^27^

3-Methyladenine (3-MA) is the most commonly used inhibitor of autophagy, which acts in order to inhibit the early stage of the pathway by blocking hVPS34. Previous studies performed on ATRA-differentiated leukemia cells^10,20^ or GM-CSF-differentiated monocytes^22^ used 3-MA in the concentrations of 5 and 10 mM. As shown in Figure 2b, the same concentrations of 3-MA decreased the number of viable cells and abolished differentiative effects of AICAR, ATRA and PMA.

**Figure 3:**
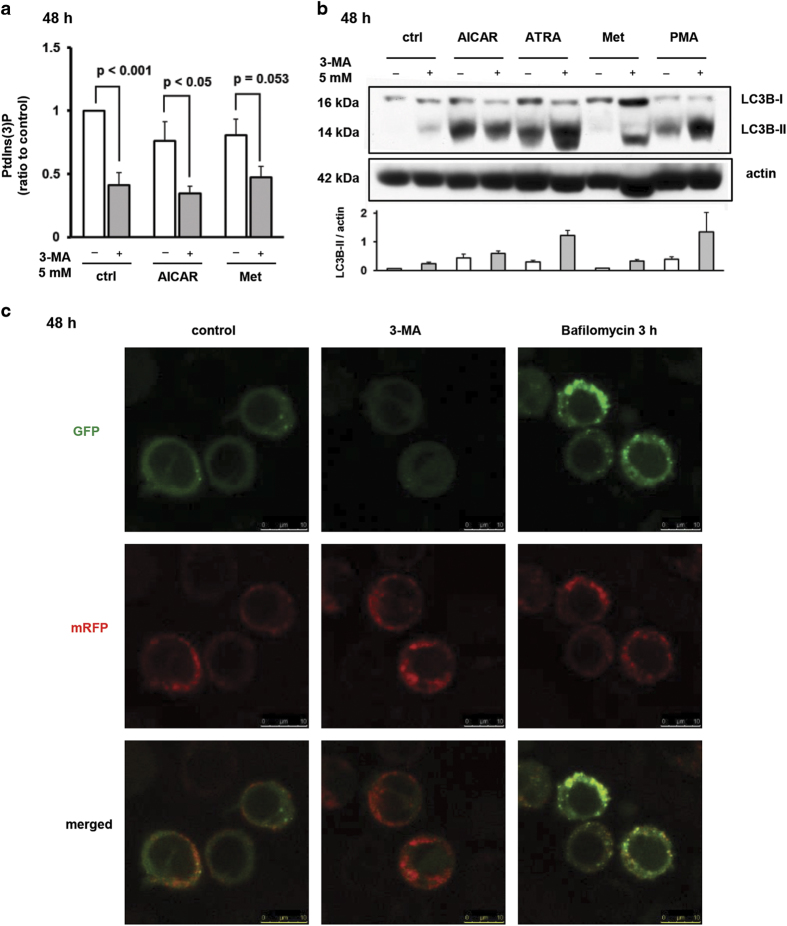
PI3K class III (hVPS34) inhibitor 3-MA reduces PtdIns(3)P but increases the levels of LC3B-II and autophagic flux. (**a**) U937 cells were treated with AICAR (0.5 mM) or metformin (15 mM) for 48 h and lipids were extracted by chloroform–methanol procedure. The levels of PtdIns(3)P were determined using Mass ELISA assay. Results are mean±S.E.M. of three independent experiments performed. (**b**) U937 cells were incubated with AICAR (0.5 mM), ATRA (1 *μ*M), metformin (15 mM) and PMA (50 nM). 3-MA (5 or 10 mM) was added 15 min before addition of agents tested. Total cell lysates were isolated 48 h after addition of agents and analyzed by western blot. Representative immunoblot from three independent experiments is shown. Blots were scored by densitometry and graph represents mean±S.E.M. of LC3B-II/actin ratios from three independent experiments. (**c**) U937 cells stably transfected with mRFP-GFP-LC3B were incubated with 3-MA (5mM) for 48 h or bafilomycin (50 nM) for 3 h. Representative images of confocal microscropy from two independent experiments performed in duplicates are shown. Images are ×63 with ×4 times digital zoom, scale bar=10 *μ*m.

Although used as an inhibitor of autophagy, 3-MA is not highly specific for hVPS34 as it inhibits both PI3K class III (hVPS34) and PI3K class I (PI3KC1). Two classes of PI3K enzymes have opposite effects on autophagy; the activation of hVPS34 induces, and the activation of PI3KC1 inhibits autophagy. Recent study reported stimulatory effects of 3-MA on autophagy in cells that were incubated in medium containing all nutrients, and these effects were explained by differential temporal effects of 3-MA on hVPS34 and PI3KC1.^28^ To check whether the presence of 3-MA for 48 h efficiently decreased the activity of hVPS34 in our model, the level of PtdIns(3)P was measured by commercial Mass ELISA Assay. As shown in Figure 3a, the presence of 5 mM 3-MA significantly decreased the level of PtdIns(3)P in control cells and cells treated with AICAR for 48 h.

Once we proved that the activity of hVPS34 was significantly inhibited after 48 h of treatment with 3-MA, the effect of the inhibitor on autophagy was determined by measuring the level of LC3B-II. As shown in Figure 3b, 48 h incubation of U937 cells with 3-MA increased the accumulation of LC3B and the levels were further increased in cells treated with combination of agents and 3-MA. Finally, additional experiments were performed on U937 cells stably transfected with plasmid containing mRFP-GFP-LC3B. As shown in Figure 3c, the punctate pattern of 3-MA-treated cells was different than the pattern observed in bafilomycin-treated cells, suggesting that an increase in the level of LC3B in 3-MA-treated cells is due to an increase in autophagy flux. Therefore, we concluded that the inhibitory effects of 3-MA on the expression of differentiation markers in our model were not due to an inhibition of autophagy.

**Figure 4:**
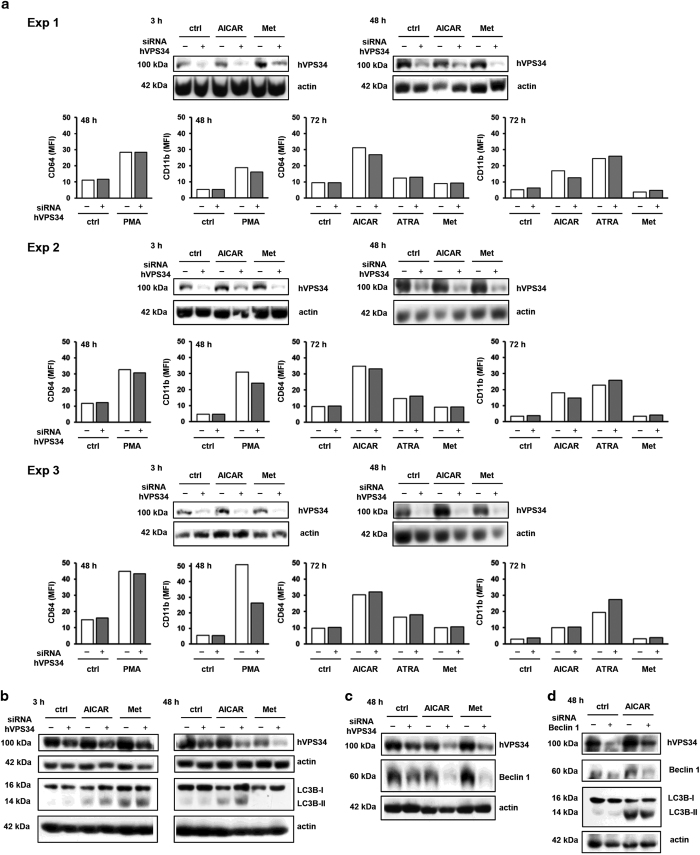
Normal levels of hVPS34 are not required for AICAR-mediated differentiation of U937 cells. (**a**) Cells were transfected with siRNA against hVPS34 and respective non-targeting siRNA was used as a negative control. AICAR (0.5 mM), ATRA (1 *μ*M), metformin (15 mM) or PMA (50 nM) were added 24 h after transfection. Total cell lysates were isolated 3 or 48 h after addition of agents and analyzed by western blot. The expression of differentiation markers was analyzed by flow cytometry 48 or 72 h after addition of agents. Western blot analyses and data obtained by flow cytometry are shown for each of the three independent experiments (Exp 1–3). (**b**, **c**) Cells were transfected with siRNA against hVPS34 or respective non-targeting siRNA. AICAR (0.5 mM) or metformin (15 mM) were added 48 h after transfection. Total cell lysates were isolated 3 or 48 h after addition of agents and analyzed by western blot for the expression of LC3B-II (**b**) or Beclin 1 (**c**). Representative images of at least three independent experiments are shown. (**d**) Cells were transfected with siRNA against Beclin 1 or respective non-targeting siRNA. AICAR (0.5 mM) was added 48 h after transfection. Total cell lysates were isolated 48 h after addition of AICAR and analyzed by western blot. Representative images of at least three independent experiments are shown.

Although our findings ruled out the possible role of 3-MA as a specific inhibitor of autophagy, the correlation between low level of PtdIns(3)P and the inhibitory effect on the expression of differentiation markers prompted us to further elucidate the possible role of hVPS34 by siRNA-mediated downregulation. Figure 4a shows the results of three independent experiments in which the exponentially growing cells were transfected by siRNA and plated 24 h after transfection in the presence of agents. The efficiency of downregulation was tested 3 and 48 h after addition of agents by western blot, and the expression of CD64 and CD11b 48–72 h after addition of agents. As shown in Figure 4a, a decrease in the level of hVPS34 had no effects on AICAR- and ATRA-mediated increase in the expression of markers. In some experiments, cells transfected with siRNA targeting hVPS34 had lower expression of CD11b upon stimulation with PMA.

It is difficult to estimate the effects of hVPS34 downregulation on autophagy, since different effects of the lack of the hVPS34 have been reported on the expression of LC3B as an autophagy marker,^29^ including even an increase in LC3-II due to decreased autophagic degradation.^30^ When we tested the effects of hVPS34 down-modulation in U937 cells, no change in the expression of LC3B was detected in cells after transfection (Figure 4b).

Canonical or classical autophagy pathway that is activated during starvation includes the formation of complex of hVPS34 with protein Beclin 1.^8^ We next tested for the level of Beclin 1 in cells transfected with hVPS34 siRNA. As shown in Figure 4c, a decrease in the level of Beclin 1 was associated with a decrease in the level of hVPS34. Although the level of LC3B-II was slightly decreased in AICAR-treated cells (Figure 4d), a decrease in the level of hVPS34/Beclin 1 proteins had no effects on the increase in the expression of CD11b and CD64 induced by AICAR and ATRA (data not shown).

**Figure 5:**
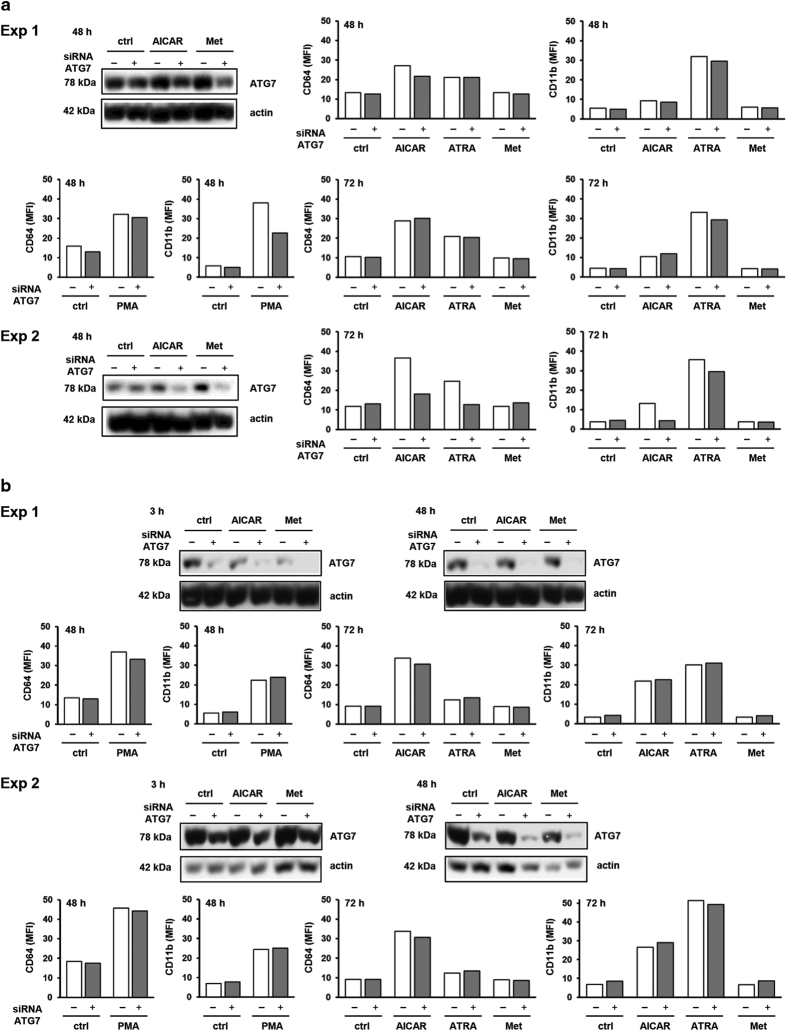
The effect of decrease in Atg7 protein levels on the expression of differentiation markers. (**a**) Cells were transfected with siRNA against Atg7 (one sequence) and respective non-targeting siRNA. AICAR (0.5 mM), ATRA (1 *μ*M), metformin (15 mM) or PMA (50 nM) were added 24 h after transfection. Total cell lysates were isolated 48 h after addition of agents and analyzed by western blot. The expression of differentiation markers was analyzed by flow cytometry 48 or 72 h after addition of agents. Western blot analyses and data obtained by flow cytometry are shown for each of the two independent experiments (Exp 1–2). (**b**) Cells were transfected with siRNA against Atg7 (four sequences) and respective non-targeting siRNA. AICAR (0.5 mM), ATRA (1 *μ*M), metformin (15 mM) or PMA (50 nM) were added 24 h after transfection. Total cell lysates were isolated 3 or 48 h after addition of agents and analyzed by western blot. The expression of differentiation markers was analyzed by flow cytometry 48 or 72 h after addition of agents. Western blot analyses and data obtained by flow cytometry are shown for each of the two independent experiments (Exp 1–2).

In accordance with the current guidelines,^16^ it is necessary to verify the autophagy dependence of a phenotype by knocking down several different *ATG* genes. In other Beclin 1-independent models, the level of LC3B was shown to depend on the expression of ATG7.^31^ Therefore, U937 cells were next transfected with 28 nM siRNA containing one sequence specific for ATG7. As shown in Figure 5a, a significant decrease in the level of ATG7 protein was paralleled with a decrease in the expression of differentiation markers in cells treated with AICAR and ATRA.

Once we observed a correlation between low level of ATG7 protein and inhibition of AICAR and ATRA-mediated increase in the expression of CD64 and CD11b, we tried to confirm that a phenotype results from specific siRNA-induced silencing of *ATG7* gene and not from potential off-target effects of individual siRNA. Pooling of multiple siRNAs to the same target have been suggested to reduce off-target silencing.^32^ Therefore, we tried to reproduce a phenotype using four different autophagy-inhibiting siRNAs to the same *ATG7* gene. As shown in Figure 5b, although transfection with pool of four siRNAs significantly decreased the level of ATG7 in two independent experiments, no effects of ATG7 down-modulation on the expression of CD64 and CD11b were observed in cells differentiated in the presence of agents.

**Figure 6:**
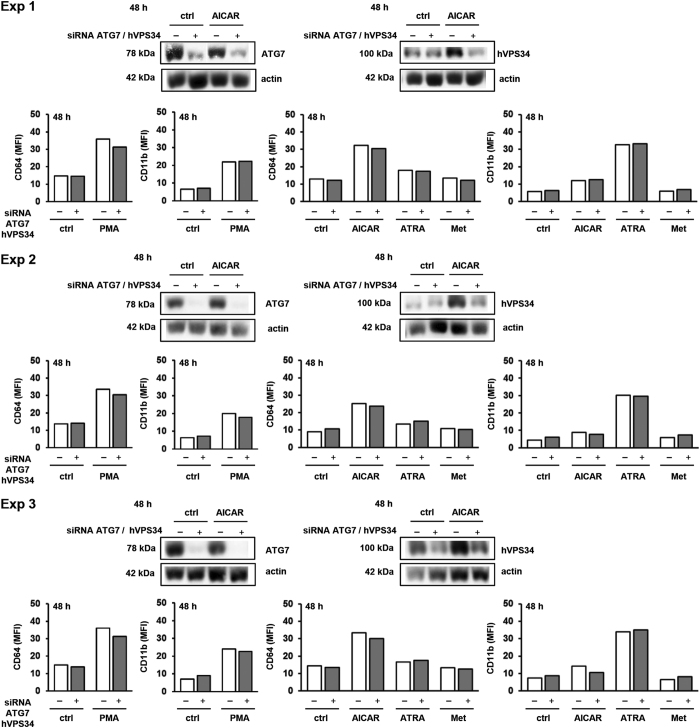
The effect of simultaneous decrease in hVPS34 and Atg7 protein levels on the expression of differentiation markers. Cells were transfected with siRNA against hVPS34 and Atg7 (four sequences) and respective non-targeting siRNA was used as a negative control. AICAR (0.5 mM), ATRA (1 *μ*M), metformin (15 mM) or PMA (50 nM) were added 24 h after transfection. Total cell lysates were isolated 48 h after addition of agents and analyzed by western blot. The expression of differentiation markers was analyzed by flow cytometry 48 h after addition of agents. Western blot analyses and data obtained by flow cytometry are shown for each of the three independent experiments (Exp 1–3).

Finally, U937 cells were simultaneously transfected with siRNA targeting hVPS34 and ATG7 and agents were added again 24 h after transfection. As shown in Figure 6, although the levels of ATG7 and hVPS34 were decreased in lysates 48 h after addition of agents, no differences in the expression of markers were observed in cells treated with differentiation agents.

## Discussion

Our previous study showed that AICAR-mediated effects on the cell viability and the expression of differentiation markers occurred independently of the level of siRNA-downregulated AMPK. Both AICAR and metformin were shown to increase the phosphorylation of AMPK on Thr172, which was used as a marker of AMPK activation, and decreased the phosphorylation of p70 S6K, a marker of mTOR inhibition, but metformin had no effects on the expression of differentiation markers.^5^ Results of our present study show that AICAR-mediated effects cannot be mimicked by specific AMPK-activator A769662, which further corroborates the hypothesis that AICAR-mediated effects are AMPK-independent. These results are in agreement with increasing number of studies showing that the majority of agents that are commonly used as AMPK agonists, including metformin and AICAR, display AMPK-independent effects on cell proliferation, metabolism and differentiation.^3,4,7,33^

Results of the present study demonstrate that AICAR and other differentiation agents increase autophagy as measured by an increase in the level of LC3B-II. Furthermore, all data obtained with bafilomycin A1 or mRFP-GFP-LC3 proved that the increase in the expression of LC3B-II was due to an increase in the autophagy flux, and not to a decrease in lysosomal degradation. Again, different effects of AICAR and metformin on autophagy flux further suggest that their effects are AMPK-independent. Although the activation of AMPK and inhibition of mTOR are well known inducers of autophagy through activation of ULK1 and hVPS34,^8^ several studies demonstrated that autophagy can occur in the absence of AMPK in response to pharmacological AMPK modulators.^7,34,35^ The effects of AICAR on the increase of LC3B-II in U937 cells correlates well with the effects of other differentiative agents in U937 cells, and the effects of both ATRA and PMA on the level of LC3B-II are similar to the ones previously described in NB4^(refs 10,11,12)^ or K562^(ref. 14)^ cell lines. In NB4 cells, the role of autophagy in differentiative effects of ATRA was proposed based on the inhibitory effects of 3-MA on the expression of CD11b.^10^ Although both chloroquine and 3-methyladenine prevented AICAR-mediated increase in the expression of CD11b and CD64 in our model, we cannot conclude that autophagy is necessary for differentiative effects. Our additional studies showed that 3-MA actually increased the level of LC3B-II, even when applied at concentration and time interval that efficiently inhibited the activity of hVPS34. Therefore, the present study further corroborate the hypothesis that 3-MA cannot be used as a specific pharmacological inhibitor of autophagy, at least not under nutrient-rich conditions.^28^

In the classical autophagy pathway, the key initiation signal is provided by hVPS34/Beclin 1 complex, and the elongation of autophagosome is dependent on ATG7.^8^ The literature search reveals many studies in which the selection of various autophagy knockdown targets in leukemia cell lines has different effects on differentiation, and the simplest explanation for these findings could be that the role of autophagy in leukemia cell line may be cell type and/or agonist dependent. In K562 cells, shRNA-mediated knockdown of ATG7 increased CD71 and glycophorin as markers of erythroid differentiation,^19^ but in NB4 cells the lack of ATG7 protein inhibited ATRA-mediated increase in CD11b.^20^ The downregulation of Beclin 1 by siRNA moderately, but significantly, inhibited vitamin D_3_-mediated increase in CD14 in HL-60 cells.^13^ In NB4 cells, shRNA-mediated downregulation of hVPS34 inhibited the expression of CD11b,^12^ but downregulation of Beclin 1 had no effects on ATRA-mediated increase in CD11b^12^ or CD11c.^11^ The effects of the downregulation of the same protein correlate better with the marker tested than with the mode of downregulation (shRNA versus siRNA) since downregulation of p62/SQSTM1 by shRNA^36,37^ inhibited ATRA-mediated increase in CD11b, but had no effects on ATRA-mediated increase in CD11c.^17^

There are no reports regarding the role of autophagy in differentiation of U937 cells, but the role of autophagy was investigated in differentiation of primary monocytes into macrophages. In CSF-1-stimulated monocytes, siRNA for Beclin 1, ATG7 and ATG5 decreased both the level of LC3B and CSF-1-mediated increase in CD71 and CD163.^38^ However, in contrast to AICAR-mediated effects in U937 cells, CSF-1-mediated effects on differentiation and autophagy were AMPK-dependent.^39^ Current guidelines in autophagy research suggest focusing on *in vivo* models instead of cell lines.^40^ The analyses of Atg7^f/f^;Vav-Cre mice lacking Atg7 in hemopoietic system revealed severe anemia and lymphopenia and self-renewal defects in hemopoietic stem cells (HSC), but the number of CD11b-positive cells was actually increased resembling myelodysplastic syndrome.^41,42^ An extensive analysis of macrophages of Atg7^f/f^;Vav-Cre mice revealed a modified response to LPS and IFN*-γ*-stimulation, but no differences in the expression of CD11b were detected, and the level of CD64 was not investigated.^43^ The analysis of Atg7^f/f^;Lyz-Cre mice in which the Atg7 deletion was limited to myeloid cell lineage revealed normal number of myeloid cells and normal physiologic induction of monocyte to macrophage differentiation. However, in contrast to HSC from Atg7^f/f^;Vav-Cre mice in which Atg7 deletion impairs autophagy, Atg7-deficient cells of Atg7^f/f^;Lyz-Cre mice maintain an active Atg7-independent alternative autophagy that depended on *Rab9*.^44^

Although results of our study suggest that canonical autophagy is not necessary for differentiation induced by AICAR and ATRA in U937 cells, we cannot completely rule out the possibility that minimal levels of Atg7, Vps34 or Beclin 1 in siRNA-treated cells are sufficient to allow differentiation to proceed. In comparison to other AML cell lines, U937 cells have higher activity of Akt and mTOR, probably due to heterozygous deletion of phosphatase and tensin homolog (PTEN),^45,46^ and loss of PTEN has been reported to inhibit autophagy without affecting LC3 lipidation,^47^ which raises a possibility that even low level of autophagy in U937 cells may be sufficient for differentiation to occur. Another possibility is that an increase in lipidated LC3B is not necessary for the expression of surface markers, but occurs simultaneously during differentiation since LC3B has been reported in both LC3B-associated phagocytosis and antigen presentation.^48^ Models of oncogene-transformed cell lines grown under nutrient-rich conditions and exposed to different agents are obviously different from a simple model of starving yeast cells in which principal members of canonical autophagy pathway, were initially described. In last years, several studies have reported that many inducers of autophagy flux act independently from Beclin 1/Vps34 complex, especially inducers of either differentiation or apoptosis.^11,12,31,49^

In conclusion, results of our study show that AMPK-independent effects of AICAR-mediated differentiation include induction of autophagy flux that is common to other inducers of differentiation, including ATRA and PMA. However, the normal levels of key proteins of canonical autophagy pathway, including Beclin 1, hVPS34 and Atg7, are not necessary for differentiation of U937 cells in response to AICAR.

## Materials and methods

### Chemicals

AICAR (A9978), 3-methyladenine (M9281) and chloroquine (C6628) were purchased from Sigma (St Louis, MO, USA) and dissolved in sterile water to stock concentrations of 100, 200 and 50 mM, respectively. 1,1-Dimethylbiguanide hydrochloride (metformin, D150959), PMA (P8139) and bafilomycin A1 (B1793) were obtained from Sigma and dissolved in RPMI-1640 medium, 100% dimethylsulfoxide (DMSO) and ethanol to stock concentrations of 1 M, 50 *μ*M and 100 *μ*M, respectively. ATRA (#554720) and rapamycin (#553210) were purchased from Calbiochem (San Diego, CA, USA) and dissolved in DMSO to stock concentrations of 1 mM and 20 *μ*M, respectively. Anti-CD11b-FITC (IM0530) and anti-CD64-FITC (IM1604), FITC-conjugated mouse immunoglobulin 1 (IgG1) (IM0639) were purchased from Immunotech Beckman Coulter (Marseille, France). Cell lysis buffer (#9803), antibodies against LC3B (#3868), p62/SQSTM1 (#5114), Ser555 p-Ulk1 (#5869), Beclin 1 (#3495), PI3 Kinase Class III (#4263), Atg7 (#8558) anti-rabbit IgG (#7074) and anti-mouse IgG (#7076) conjugated to horseradish peroxidase were purchased from Cell Signaling Technology (Beverly, MA, USA). Enhanced chemiluminescence (ECL) substrate was obtained from Thermo Fisher Scientific (Waltham, MA, USA), and protein assay from Bio-Rad Laboratories (Hercules, CA, USA; #500-0006) or Sigma (B6916). A Neon Transfection System 100 *μ*l Kit (#MPK10096) and anti-rabbit IgG secondary antibody conjugated to Alexa Fluor 488 (A11034) were purchased from Invitrogen (Carlsbad, CA, USA). Small interfering RNA (siRNA) SignalSilence Unconjugated Control (#6568), SignalSilence Beclin 1 siRNA I (#6222) and II (#6246), SignalSilence Atg7 siRNA I (#6604) were purchased from Cell Signaling Technology. ON-TARGETplus SMARTpool Human ATG7 (L-020112-00), PIK3C3 (L-005250-00) and negative control (D-001810-10) siRNA were obtained from Dharmacon (Lafayette, CO, USA). Monoclonal anti-β-actin antibody (#A5441), propidium iodide (PI), RNaseA, Igepal, color markers, bovine serum albumin, Triton X-100, sodium dodecyl sulfate (SDS), leupeptin and phenylmethylsulfonyl fluoride (PMSF) were from Sigma, RPMI-1640, fetal bovine serum (FBS), penicillin/streptomycin were from Gibco/Invitrogen (Grand Island, NY, USA) and G418 was from Roche (Basel, Switzerland). Vectastain normal goat serum (FI-1200) and Vectashield mounting medium (H-1000) were from Vector Laboratories Inc. (Burlingame, CA, USA). Deoxyribonuclease I (D5025) was from Sigma. PtdIns(3)P Mass ELISA Kit (K-3300) was from Echelon Biosciences Inc. (Salt Lake City, UT, USA).

### Cell culture

U937 cells were obtained from two sources: one was a kind gift from Dr. Mirna Golemovic (Clinical Hospital Centre, Croatia) and another was bought from European Collection of Animal Cell Cultures (ECACC no. 88112501; Porton, Salisbury, UK). The cells were maintained in RPMI-1640 supplemented with 10% heat-inactivated FBS, 2 mM L-glutamine, 50 U/ml penicillin and 50 *μ*g/ml streptomycin at 37 °C in a humidified atmosphere containing 5% CO_2_.

For the experiments, cells were harvested, resuspended in fresh medium containing FBS, l-glutamine and penicillin/streptomycin and seeded at a concentration of 0.2×10^6^/ml in six-well plates. The cells were incubated for various time intervals in the presence of 0.5 mM AICAR, 1 *μ*M ATRA, 15 mM metformin and 50 nM PMA. Autophagy modulators, chloroquine (25 *μ*M), 3-MA (5 or 10 mM) and rapamycin (20 nM), were added 15 min prior to other agents. Bafilomycin was added for the last 3 h of incubation at a final concentration of 50 nM. At the end of incubation, the number of viable cells was determined by trypan blue staining and hemocytometry.

### Expression of surface markers

The expression of surface markers was determined by flow cytometric analysis, as previously described.^50^ Briefly, cells were collected, washed and incubated with FITC-conjugated monoclonal antibodies against CD11b and CD64, or with their isotypic control for 20 min, washed and analyzed using the FACSCalibur system and Cell Quest software (Becton Dickinson Immunocytometry Systems, San Jose, CA, USA). Live cells were gated based upon forward and side scatter patterns. A total of 15 000 events were collected for each marker from the gated area detecting viable cells. On a single fluorescence histogram of the sample stained with isotypic control, a cursor was set to include up to 1.0% of events as positive. To determine the mean fluorescence intensity (MFI) of the sample, MFI levels of isotypic controls were deducted from MFI levels of the cells stained with CD-specific antibodies.

### Isolation of total cell lysates and western blot analysis

At the end of incubation, cells were collected by centrifugation, washed in ice-cold PBS and incubated in 1× cell lysis buffer containing freshly added 1 *μ*M microcystin and 1 mM PMSF on ice. After 10 min, cells were further disrupted by seven passages through a 23-gauge needle, incubated on ice for 10 min and centrifugated at 14 000×*g* for 10 min. The supernatants were collected and stored at −80 °C. The protein concentration of each sample was determined using Bradford protein assay (Bio-Rad or Sigma).

Western blot analysis was performed as previously described.^46^ Briefly, equal amounts of proteins (35–50 *μ*g) in each sample were loaded onto two parallel 8 or 12% SDS-polyacrilamide gels. Electrophoresis was carried out using the Bio-Rad mini-Protean apparatus, and proteins were transferred to nitrocellulose membranes (Whatman, Dassel, Germany) using the Bio-Rad mini Trans-Blot system. After blocking for 30 min in TBS-Tween buffer containing 5% (w/v) non-fat dried milk, membranes were incubated with primary antibodies (1:20 000 for actin; 1:1000 for other antibodies) overnight at 4 °C, and then with secondary antibodies (1:2000) for 120 min at room temperature. Bands were visualized using the ECL kit. Relative densitometric values for autoradiography signals were analyzed with Adobe Photoshop CS version 8.0 software (San Jose, CA, USA).

### Isolation of acidic lipids and competitive PtdIns(3)P Mass ELISA

Measurement of total cellular PtdIns(3)P levels was carried out using PtdIns(3)P Mass ELISA Kit (Echelon) following the manufacturer’s instructions. Briefly, after 48 h incubation with AICAR (0.5 mM) and metformin (15 mM), viable cells were counted and the volume adjusted so that each sample contains the same number of cells (13–15×10^6^). Neutral lipids were isolated with methanol:chloroform (2:1) extraction and acidic lipids were subsequentially isolated using methanol:chloroform: 12 N HCl (80:40:1). Dried acidic lipids were stored at −20 °C. For PtdIns(3)P measurement with Mass ELISA assay, dried lipids were reconstituted in PBS with 0.05% Tween-20 and 3% Protein Stabilizer and incubated for 30 min in a water bath sonicator at room temperature. The quantities of PtdIns(3)P in each sample were calculated by comparison with a standard curve derived from measurements of PtdIns(3)P standards supplied by the manufacturer using nonlinear regression analysis (GraphPad Prism Software, La Jolla, CA, USA).

### siRNA transfection

Transfection with controls and indicated siRNAs targeting proteins of interest was performed using the Neon transfection system (Invitrogen) as previously described.^5^ Briefly, the cells were collected in their exponential growth, resuspended in transfection buffer at a concentration of 22×10^6^ cells/ml and siRNAs were added at recommended concentrations. The electroporation was carried out in a 100 *μ*l tip, with single pulse, at a voltage of 1050 V and pulse width of 50 ms. Following the electroporation, 100 *μ*l of cell suspension was resuspended in 200 *μ*l of RPMI-1640 with 10% FBS and 2 mM l-glutamine without penicillin/streptomycin, incubated for 15 min at 37 °C and resuspended in the total volume of 5 ml RPMI without antibiotics. Final concentrations of 28 nM siRNA against Beclin 1 and ATG7 (Cell Signaling Technologies) or 45–140 nM siRNA against hVPS34 and ATG7 (Dharmacon) were used. Transfected cells were incubated for 24 or 48 h, collected, resuspended in fresh medium and plated in six-well plates for the differentiation experiments. An aliquot of cells was used for the preparation of total cell lysates and the level of downmodulated proteins was determined by western blot analysis.

### Immunostaining for confocal microscopy

U937 cells were stained for LC3B in round bottom 5 ml tubes according to the manufacturer’s instructions. Briefly, cells were fixed with ice-cold 100% methanol at −20 °C, blocked in 5% normal goat serum with 0.3% Triton X-100, washed in PBS with addition of 0.1  mg/ml DNAse to prevent clumping, stained with primary antibody at +4 °C overnight (1 : 200) and with secondary antibody (1:600) at room temperature for 120 min. The pellet of immunostained cells was resuspended in 20 *μ*l Vectashield mounting medium and mounted on slides. Images were taken using a Zeiss LSM 510 Meta confocal microscope with a plan-apochromat ×63 1.4 NA oil immersion and analyzed with Zeiss LSM software (Jena, Germany).

### Generation of stably transfected U937 cell line expressing mRFP-GFP-LC3B

ptfLC3 (mRFP-GFP-LC3) plasmid was a gift from Tamotsu Yoshimori (Addgene plasmid # 21074, Cambridge, MA, USA). U937 cells (2×10^6^) were transfected with 10 *μ*g of linearized and purified plasmid using the Neon Transfection system as described above. Cells stably expressing mRFP-GFP-LC3 were selected in medium supplemented with 400 *μ*g/ml G418 during 2 weeks. After selection, cells were expanded and maintained in medium containing 250 *μ*g/ml G418.

For fluorescence microscopy, cells were seeded on chambered coverslips (Nunc Lab-Tek II) with pharmacological agents of interest. Images were acquired on a Leica TCS SP2 AOBS confocal microscope using a plan-apochromat ×63 1.4 NA oil immersion with ×4 digital zoom and images were analyzed with ImageJ software (Bethesda, MD, USA) and Adobe Photoshop CS version 8.0 software.

### Statistical analysis

Data are presented as mean±standard error of the mean (S.E.M.) from the number of experiment indicated. Difference between two groups was determined by Student’s *t*-test and *P-*values <0.05 were considered statistically significant.

## Additional information

**Publisher’s note:** Springer Nature remains neutral with regard to jurisdictional claims in published maps and institutional affiliations.
